# Sarcopenia in atrial fibrillation: a prospective study

**DOI:** 10.55730/1300-0144.6136

**Published:** 2025-10-26

**Authors:** Mehmet Akif TOPÇUOĞLU, Ezgi YILMAZ, Doğan Dinç ÖGE, Ali Naim CEREN, Rıdvan Muhammed ADIN, Ethem Murat ARSAVA

**Affiliations:** 1 Department of Neurology, Faculty of Medicine, Hacettepe University, Ankara, Turkiye; 2 Faculty of Physical Therapy and Rehabilitation, Hacettepe University, Ankara, Turkiye

**Keywords:** Sarcopenia, muscle health, muscle ultrasound, malnutrition, physical activity, heart failure

## Abstract

**Background/aim:**

The link between atrial fibrillation (AF) and lean body mass is unclear. This study presents the effects of AF on sarcopenia indices, including ultrasonographic muscle architecture.

**Materials and methods:**

In this study conducted in neurology units, 72 subjects with AF (mean age: 71 ± 11years, 49% female) were compared with 538 without AF (mean age:66±12years; 53% female) in terms of sarcopenia indices [anthropometry; Short Physical Performance Battery (SPPB); hand grip strength (HGS); bioimpedance analysis (BIA) indices; skeletal muscle mass index (SMMI) normalized to weight, height and body mass index (BMI); and phase angle (PhA)]; muscle ultrasonography [thickness, cross-section area (CSA), fiber length (fL) and pennation angle (PeA) of rectus femoris (RF), biceps brachii (BB), vastus lateralis (VL), vastus intermedius (VIM) and gastrocnemius medialis (GCM)]; possible causes of sarcopenia such as malnutrition; and consequences of sarcopenia [fall, fatigue, SarQol] along with sarcopenia screening (SARC-F). Possible associations were tested in multiple exploratory linear models, and partial r(pr) and p were reported.

**Results:**

Participants with AF exhibited significantly higher SARC-F and SarQol scores. In multivariable models adjusted for age, sex, height, and body weight, AF was independently linked to lower mean and maximum HGS (pr = −0.137, p = 0.014; pr = −0.130, p = 0.02), reduced PhA (pr = −0.193, p < 0.001), decreased RF thickness (pr = −0.120; p = 0.004), and diminished RF+VIM thickness (pr = −0.098; p = 0.019). Additional muscle ultrasound parameters, including BB-CSA, RF-CSA, BB-brachialis thickness, VL fL, and PeA and BIA indices, including SMMI(height), SMMI(weight), and SMMI(BMI), tended to be lower in AF.

**Conclusion:**

AF is linked to impaired muscle health and reduced quality of life. Management should include thigh muscle ultrasound and strategies to prevent malnutrition.

## 1. Introduction

Sarcopenia is a progressive and generalized skeletal muscle disorder characterized by the loss of muscle mass, strength, and function. Initially recognized as a geriatric syndrome, it is now understood to affect a broader population, including individuals with chronic diseases, sedentary lifestyles, and metabolic dysfunction. The condition is associated with adverse outcomes such as frailty, falls, disability, and increased mortality, and has been formally classified by the World Health Organization under ICD-10 [1].

Atrial fibrillation (AF), the most common sustained cardiac arrhythmia, shares several pathophysiological features with sarcopenia, including systemic inflammation, oxidative stress, and age-related degeneration. Both conditions are prevalent in older adults and often coexist with multimorbidity, complicating clinical management and prognostication.

Emerging evidence suggests a potential bidirectional relationship between AF and sarcopenia. On one hand, sarcopenia may predispose individuals to AF through mechanisms such as autonomic imbalance, impaired metabolic reserve, and altered body composition. On the other hand, AF may accelerate muscle wasting via reduced perfusion, neurohormonal activation, and physical inactivity, especially when accompanied by heart failure [2–5].

Despite these plausible links, the literature remains inconclusive. Studies vary widely in their definitions of sarcopenia, assessment methods [e.g., dual-energy X-ray absorptiometry (DXA), bioimpedance analysis (BIA), handgrip strength], and population characteristics.[6] Some report lower lean body mass in AF patients, while others find elevated levels, possibly reflecting fluid retention or obesity-related confounding.[2,4,7] Notable features of the sarcopenia-AF-AF relationship include a rising incidence of sarcopenia in individuals with AF, particularly in the context of heart failure and its increasing severity [8,9]. Impaired muscle health has also been associated with elevated in-hospital and all-cause mortality among AF patients [6,10]. Furthermore, obesity and body weight variability appear to contribute to the heightened prevalence of sarcopenia in this population [5,11,12]. Moreover, sarcopenic obesity, a phenotype marked by concurrent muscle loss and excess adiposity, has been increasingly recognized as a high-risk profile for AF development and poor outcomes [3,13].

To address these gaps, we conducted a cross-sectional study comparing individuals with and without AF, aiming to determine the frequency, phenotypic characteristics, and clinical consequences of sarcopenia in this population.

## 2. Methods

### 2.1. Subjects

A total of 610 individuals (mean age: 66.9 ± 12.4 years; 52.5% female) were recruited from neurology outpatient clinics and inpatient services between October 2018 and May 2021 and enrolled in the study. The study population included 428 patients with various neurological conditions (304 patients with stable neurovascular disease, 87 with dementia, 24 with Parkinson’s disease (PD), 11 with multiple sclerosis (MS), and one with stroke plus dementia and another one with stroke plus PD. Additionally, 182 individuals without any neurological disease were included. Subjects suffering from neurological or orthopedic conditions that impair gait and cause significant motor deficits, with cardiac pacemakers, decompensated heart failure, and renal failure were excluded from the study. Written consent was obtained from all persons and, where necessary, from their relatives for each study procedure. Study protocols were approved by the ethics committee of Hacettepe University (Decision numbers: GO 22/427 for Prospective stroke database; GO 21/469 and GO 18/569 for AF and sarcopenia). The diagnosis of AF was established based on clinical history and electrocardiogram.

### 2.2. Demographic, clinical, and anthropometric data

Age, sex, educational status (mean education years: 9.4 ± 4.7), atherosclerosis risk factors, all diseases and medicines used, including hypertension, diabetes mellitus (DM), dyslipidemia, coronary artery disease, heart failure, rheumatic valve disease, and prosthetic heart valve replacement were recorded. The Chapman and Chapman test was used for hand dominance determination [14]. The duration of the existing neurological diseases, the side of the disease, if any, and the severity of the disease were determined. For the latter, the Expanded Disability Status Scale (EDSS) [15] was used for MS (mean EDSS: 2.4 ± 2.3), the modified Hoehn and Yahr scale (mHYS) [16] for PD (mean mHYS: 2±1), and the Mini-Mental Status Examination (MMSE) score [17] for dementia/cognitive impairment (mean MMSE: 23.6 ± 7.4). Height, weight, body mass index (BMI), upper middle arm circumference (UMAC), calf circumference, thigh circumference, waist circumference, and hip circumference were measured in all.

### 2.3. Clinical tests for sarcopenia, physical activity, and frailty

SARC-F (Strength, assistance with walking, rising from a chair, climbing stairs, and falls) [18,19] and SarQoL questionnaires [20,21] were performed in all, along with the Short Physical Performance Battery (SPPB, ≤8 abnormal) [22]. Fall Efficacy Scale [23,24], Morse Fall Scale [25,26], Barthel Index for activities of daily living (ADL) [27,28], Lawton and Brody Instrumental Activities of Daily Living (ADL) Scale [29,30], Fatigue Severity Scale [31,32], and Beck Depression Inventory [33,34] were determined for all subjects. In addition, the Malnutrition Universal Screening Tool (MUST) [35], Functional Oral Intake Scale (FOIS) [36], and the Eating Assessment Tool (EAT-10) [37] were obtained for all.

### 2.4. Hand grip strength test

Hand grip strength (HGS) test was performed with a Takei (T.K.K.5401 GRIP-D) handheld dynamometer using the standardized technique defined by the European Working Group on Sarcopenia in Older People (EWGSOP) [38]. The subjects were asked to grasp and squeeze the dynamometer in a sitting position, with their forearms on the chair armrest and their thumbs facing up. Starting from the dominant side, with 30 s of rest between each measurement, three measurements were alternately recorded from each hand. The mean of all measurements [HGS(mean)] and the highest value [HGS(max)] were included in the analyses. HGS < 32 kg for males and <22 kg for females were reported as cut-off values for the Turkish population [39].

### 2.5. Bioimpedance analysis (BIA)

Muscle mass indices were measured with a multifrequency segmental body bioimpedance analyzer (BIA; Tanita MC-780 MA model and The Bodystat QuadScan 4000) Briefly, measurements were performed with a total of eight electrodes at frequencies of 5 kHz, 50 kHz, and 250 kHz, 4 of which were on the platform where the subject was standing and four on the apparatus held by them. Measurements were performed when the subjects were fasting for at least 3 h, had not engaged in heavy exercise in the last 12 h, and did not need to urinate. Appendicular skeletal mass (ASM), ASMI (appendicular skeletal mass index), and SMMI (normalized for weight, height, and BMI) were detected. Lean body mass (FFM) was measured by the device. Total skeletal muscle mass (SMM) was calculated as 0.566 × FFM. Other indices and their formulas were ASMI=ASM/height^2^, SMMI(weight)=SMM/weightx100; SMMI(height)=SMM/height^2^, SMMI(BMI)=SMM/BMI, and phase angle(PhA). Cut-off values for sarcopenia were <15 kg (female) and <20 kg (male) for ASM [40], <5.5 kg/m^2^ (female) and 7 kg/m^2^ (male) for ASMI [40], <7.4–8.9 kg/m^2^ (female) and <9.1–10.8 kg/m^2^ (male) for SMMI(height) [39,41], >33.6–33.2% (female) and <37.4–40.6% (male) for SMMI(weight) [39,41], and <0.677–0.823 (female) and <1.017–1.049 (male) for SMMI(BMI), PhA (<5 abnormal) [39,41].

### 2.6. Muscle ultrasound

Recordings were performed with the 7–12 MHz linear transducer of the GE Logiq P6 ultrasound system [General Electric Medical Systems, Waukesha, WI, USA], with the patient lying supine and the head in a neutral, low semi-Fowler position. The study was performed by a neurosonologist [MAT] with over 20 years of experience, who was completely blind to the subjects’ diagnoses and other relevant information. Ultrasonic measurement techniques are explained in detail in the Supplementary Figures 1–4.

Measurements obtained with ultrasound were (i) muscle thickness: BB muscle (BB-MT); anterior arm muscles (biceps + brachialis) (Arm-MT); rectus femoris (RF vertical thickness: RF-MT), anterior thigh muscles (RF+VIM) (thigh muscle thickness: TMT), and VL (transverse, VL-MT); (ii) subcutaneous adipose tissue thickness: arm (arm-SAT) and thigh (thigh-SAT); (iii) muscle cross-sectional area: BB (BB-CSA) and rectus femoris (RF-CSA); (iv) the PeA: VL (VL-PeA) and GCM (GCM-PeA); (v) The lengths of muscle fascicles: VL (VL-fL) and GCM (GCM-fL).

### 2.7. Statistics

All data were presented as mean ± standard deviation, median (minimum–maximum), or percent as appropriate. Distribution normality was examined using histogram and Q-Q plot visual analyses, and the Shapiro–Wilk and Kolmogorov–Smirnov tests. Student’s t, Mann–Whitney U, or analysis of variance (ANOVA) tests were used for numerical values, and chi-square or Fisher’s exact tests were used for categorical variables. MMSE score. Spearman’s or Pearson’s methods were used for correlation analysis, as appropriate. Possible associations were tested in multiple exploratory linear models. Models were created separately for AF and sarcopenia parameters, HGS(max), HGS(mean), SPPB, SMMI(height), SMMI(weight), SMMI(BMI), PhA, ASM, BB-MT, Arm-MT, BB-CSA, RF-CSA, RF-MT, TMT, VL-MT, VL-FL, VL-PA, GCM-MT, GCM-FL, and GCM-PA values were adjusted according to age, sex, height, and weight, and aR^2^, B, r (partial), and p-values of the models were reported. A p-value less than 0.05 was accepted for statistical significance. The Statistical Package for Social Sciences (SPSS) version 22.0 (SPSS Inc., Chicago, Illinois, USA) program was used for all analyses.

## 3. Results

The mean age of 72 subjects with AF is significantly higher than that of 538 subjects without AF [70.9 ± 10.5 (44 to 90) vs. 66.2 ± 12.4 (23 to 92) years, p = 0.002]. Sex distribution and educational status (8.4 ± 5.6 vs. 9.6 ± 4.7 years, p = 0.052) are equal in groups with and without AF. Hypertension and dyslipidemia are significantly higher in AF, while DM shows a nonsignificant increase. Coronary artery disease, heart failure, rheumatic valve disease, and prosthetic heart valve disease were more common in patients with AF, as expected (Table 1A). The prevalence of AF was found to be 14.1% in neurovascular patients, 13.7% in healthy participants, 4.2% in PD patients, and 3.4% in patients with dementia. The neurological disease diagnoses, duration, severity, and laterality (if applicable) did not differ significantly between patients with and without AF (data were not presented).

Average body weight was 3.8 kg and BMI 1 unit higher in patients with AF, although not reaching statistical significance (77.9 ± 17 vs. 74.1 ± 14.8 kg, p = 0.051; and 29.8 ± 5.5 vs. 28.8 ± 6.2 kg/m^2^, p = 0.184). Waist, hip, thigh, calf, and arm circumferences were slightly higher in AF but not statistically different. Of note, there was no difference in the extremities in terms of right and left, dominant and nondominant sides (Table 1B).

Although it was numerically lower in patients with AF, no statistically significant difference in muscle strength or physical performance was observed (Table 1C, D). The proportion of those with an SPPB score of eight or less was 24% in AF and 20% in those without AF (p = 0.604). Patients with AF had significantly lower quality of life, with a SarQoL total score of 64.7 ± 21.5 (28.6 to 100) compared with 75.4 ± 18.0 (26.7 to 100) in those without AF (p = 0.002). They also reported greater fear of falling, as reflected in Fall Efficacy Scale scores approximately 10 points lower (p = 0.018). Additionally, depressive symptoms were more pronounced, with Beck Depression Inventory scores about 4 points higher in the AF (p = 0.025). (Table 1E). In addition, the proportion of subjects with fatigue was significantly higher in the AF group (56% vs. 33%, p = 0.009). In contrast, the mean fatigue severity score did not differ between the groups with and without AF. No difference was detected between the groups in terms of the mean Morse Fall Scale score, the proportion of patients in the high-risk category according to the Morse Fall Scale (9% vs. 5% in patients with and without AF, respectively, p = 0.557), the average Barthel’s score, the proportion of people classified as dependent per Barthel’s score (31% vs. 26% in patients with and without AF, respectively, p = 0.527), and the average Lawton–Brody ADL score (Table 1E).

From the nutritional standpoint, AF appeared to be associated with an increased risk of malnutrition. Although not statistically significant, individuals with AF had nearly double the average MUST score (p = 0.053, Table 1F). Similarly, the median MUST score [median (range): 0 (0–2) vs. 0 (0–3) in those with and without AF, respectively; p = 0.064] and the proportion of patients with MUST=0 (no malnutrition risk: 73% vs. 85% in AF vs. non-AF groups; p = 0.399) showed a trend toward nutritional vulnerability in AF, though without reaching statistical significance. Although the FOIS and EAT-10 scores in the AF group were numerically worse, they did not reach statistical significance (Table 1F).

### 3.1. Sarcopenia parameters

The mean SARC-F score was approximately 1 point higher in patients with AF (means: 2.5 ± 2.6 vs. 1.4 ± 1.9, medians: 2 (0–7) vs. 1 (0–8), p=0.003). The proportion of patients with a SARC-F of 4 or higher was more than twice as high in patients with AF (14% vs 30%, p=0.018) (Table 2A).

The cross-sectional area of the biceps brachii muscle is approximately 1 cm^2^ smaller in patients with AF (p = 0.011). There was no difference in the vertical thicknesses of the anterior muscle (biceps+brachialis), the whole arm muscle, and only the BB muscle. Arm subcutaneous adipose tissue thickness was significantly higher in patients with AF (approximately 0.7 mm higher) (Table 2B).

Patients with AF had significantly smaller RF vertical thickness (9.7 ± 2.4 (4 to 13.45) vs 10.9 ± 2.9 (3.2 to 27.5), mm, p = 0.002) and thigh muscle thickness (17.4 ± 5.7 (9.8 to 24.75) vs.19.3 ± 5.6 (7.94 to 35.55), mm, p = 0.012), while the RF cross-sectional area only tended to be lower (p = 0.054). Thigh subcutaneous adipose tissue thickness did not differ between individuals with and without AF (p = 0.492) (Table 2B).

There was no difference in the thicknesses of the VL and GCM muscles in individuals with and without AF (p = 0.215 and p = 0.703, respectively). However, several significant differences were found in the sonic architecture of the VL and gastrocnemius muscles. PeA was significantly lower in both GCM and VL muscles of patients with AF than those without AF (p = 0.014 and p = 0.030, respectively). The mean fascicle length was significantly longer for VL (p = 0.006), but the excess of GCM fL remained at the numerical level (p = 0.180).

SMMI (weight) was significantly lower in patients with AF (36.1 ± 6.1 vs. 38.2 ± 6.1, p = 0.009). No difference was found in terms of ASM, SMMI(height), and SMMI(BMI). Phase angle was also significantly decreased in patients with AF (4.6 ± 1 vs. 5.2 ± 1, p < 0.001).

### 3.2. Regression analysis

The adverse effect of AF on HGS(mean) and HGS(max) remained significant [r(part) = −0.137, p = 0.014 and r(part) = −0.13, p = 0.02, respectively], after adjusting for age, height, weight, and female sex. The effect of AF on SPPB was not significant (Figure, Supplementary Table 1A).

In the model adjusted for age, female sex, height, and weight, the effect of AF on the phase angle remained significant [r(part) = −0.193; p < 0.001]. However, only a negative numerical correlation was detected for other BIA parameters, such as SMMI (weight), SMMI (height), and SMMI (BMI). The relationship between AF and ASM is not significant (Figure, Supplementary Table 1B).

Similar modeling revealed a small but significant and independent reduction in muscle size in both upper and lower extremities in AF cases. BB cross-sectional area [r(part) = −0.119, p = 0.004], RF muscle thickness [r(part) = −0.12, p = 0.004] and RF+VIM muscle thickness [r(part) = −0.098, p = 0.019] were significantly lower in subjects with AF. There was a tendency to decrease in RF cross-sectional area, biceps, and biceps+brachialis muscle thickness (Figure, Supplementary Table 1C, D).

We found that AF had significant effects on muscle sonographic architecture in similar modellings. In patients with AF, VL fL increased [r(part) = 0.098, p = 0.027] and PeA decreased [r(part) = −0.087, p = 0.048]. Similar trends were observed in the GCM, but did not reach statistical significance (Figure, Supplementary Table 1E).

## 4. Discussion

In our study, hand grip strength (approximately three kg), BB cross-sectional area (approximately 1 cm^2^), RF muscle thickness (approximately 1.2 mm), and phase angle (0.6°) were lower in cases with AF. In addition, we detected significant disturbances in skeletal muscle sonic architecture, including increased fL and decreased PeA in the VL. Remarkably, all these changes were less pronounced in the lower leg muscles (gastrocnemius). In addition, SARC-F was higher in these patients. In summary, our findings indicate a potential association between AF and reduced skeletal muscle mass, as evidenced by clinical assessment, ultrasonographic measurements, and bioimpedance analysis. The low correlation coefficients are due to the partial and adjusted nature of the coefficients. Of note, the association between AF and the studied parameters of muscle mass loss appears independent of age, female sex, height, and weight.

In addition, our findings suggest that sarcopenia may exert clinically meaningful effects in patients with AF, as reduced SarQol, increased fear of falling, depression, and fatigue were more frequently observed in this group. We could not test the possible causes of sarcopenia in AF in detail, but we documented a potential contribution of malnutrition risk, which tended to be higher in AF.

According to our best knowledge and detailed literature search, there is no previous muscle ultrasonographic study to detect sarcopenia in patients with AF [42]. However, the presence of sarcopenia in AF has been discussed in various aspects in studies using BIA [2,7,11,13] and DeXA[4]. Lean body mass was found to be high in people with AF in some studies [2,4,7], while it was reported to be low in others [11]. Only sarcopenic obesity was associated with an increased frequency of AF in one study [13]. However, in total, it can be said that sarcopenia and AF are risk modifiers for each other. Our findings provide supportive evidence of muscle health impairment in patients with AF, complementing and extending existing data on frailty and sarcopenia.

Clinically, slower walking speed and reduced grip strength have been demonstrated previously in patients with AF [43]. Skeletal muscle health impairment is associated with disability, insufficient capacity for activities of daily living, cognitive decline, increased institutionalization, and mortality rates in patients with cardiovascular diseases, perhaps including AF [42]. We added the fear of falling, decreased quality of life, depression, and fatigue to the negative consequences. MUST scores in subjects with AF showed a modest increase in malnutrition risk, though this was not statistically significant. Exploring mechanisms linking impaired muscle health with AF, along with approaches for detecting, diagnosing, and managing sarcopenia in this population, should be prioritized in future cardiology research.

Sarcopenia is an aging-related loss of skeletal muscle mass. It is characterized by decreased muscle strength, function, and physical performance. The frequency of sarcopenia increases in the elderly with chronic diseases. These chronic diseases include chronic obstructive pulmonary disease, chronic kidney disease, and cancer. Recently, however, there has been increasing data on the increasing incidence of sarcopenia in cardiovascular diseases and vascular diseases in sarcopenia and the additive negative interaction of sarcopenia with vascular diseases [44]. Sarcopenia is associated with faster progression of cardiovascular disease and a higher risk of death, falls, and poor quality of life, especially in the elderly [42]. However, it can be said that information about the mutual connection between sarcopenia, one of these vascular diseases, and AF is still at an early stage. Evidence from longitudinal cohort studies remains inconclusive regarding the association between sarcopenia and AF. The UK-Biobank cohort demonstrated a significantly elevated risk of incident AF among sarcopenic individuals over a ten-year follow-up [45]. In contrast, the Korean Frailty and Aging Cohort Study (KFACS) did not replicate this bidirectional risk in its 2-year longitudinal analysis [46]. Nonetheless, the cross-sectional phase of KFACS did reveal a higher prevalence of AF in participants with sarcopenia [46]. Our study provides supportive evidence for the sarcopenia–AF association and contributes meaningfully to understanding the potential interplay between these conditions.

The frequency of AF increases with advancing age. However, the reason why sarcopenia is highly prevalent in AF is probably not directly explained by the fact that both are more common later in life. Many mechanisms can be proposed for the pathophysiological association between the two. First, AF and related heart failure will reduce physical activity capacity, which will negatively affect sarcopenia. Sarcopenia will also lead to poor physical function. This interaction between physical activity and exercise capacity is likely additive and extends beyond what each does separately. Secondly, sarcopenia and cardiovascular diseases, including AF, have many common pathological pathways. These include malnutrition, physical inactivity due to decreased exercise capacity, insulin resistance, and inflammation. Each has the potential to be both cause and effect, and they interact with each other. Thirdly, the effect of AF on skeletal muscle perfusion has not been studied. However, decreased perfusion in other tissues, such as the atria and brain, has been associated with AF. Due to tachycardia during exercise, muscle perfusion may be impaired, or AF may increase, especially at levels that limit functionality [47].

Screening and instrumental testing for sarcopenia may be particularly important in subjects with cardiovascular disease. Early recognition of sarcopenia is important because interventions such as exercise, nutritional deficiencies, micronutrient supplementation, and the use of special formulas may be available to reverse or delay the progression of the muscle disorder [48]. Although not investigated, alleviation of sarcopenia may ultimately affect cardiovascular outcomes. [42,47] BMI alone is not useful for screening, as many patients will have sarcopenic obesity, a particularly important phenotype among elderly heart patients. Therefore, evaluation with BIA and DeXA is recommended. Muscle ultrasound is a simple, accessible method for diagnosing sarcopenia, as shown in our study. Cardiologists already use high-resolution ultrasound systems for echocardiography, making muscle assessment both feasible and cost-effective. This overlap allows for dual-purpose imaging—cardiac and musculoskeletal—especially valuable in aging populations. Echocardiography-enabled muscle ultrasound provides a rapid, non-invasive, and scalable approach for early screening and timely diagnosis of sarcopenia within cardiology practice.

Our study has some limitations. The number of cases with AF remained low because we first recruited subjects and then diagnosed AF. We did not collect information on AF subphenotypes or detailed cardiac characteristics. A bias may be possible due to the inclusion of people with AF who can only make physical performance assessments. In addition, because we relied primarily on history and the electrocardiogram, we might have missed patients with paroxysmal AF episodes. The inclusion of both inpatients and outpatients may have contributed to clinical heterogeneity and potential selection bias. Although a considerable proportion of our cohort had mild neurological conditions, all cases were early-stage and functionally independent. Therefore, while such comorbidities may coexist with sarcopenia, their contribution in this context is likely negligible and does not confound the observed association with AF. Nonetheless, we acknowledge that this overlap may lead to interpretive ambiguity and have accordingly noted it as a limitation.

Our study has multiple strengths. First, anthropometric and physical performance measures of sarcopenia were administered in a standardized format. Ultrasonic muscle architecture was used for the first time in patients with AF. It is also a study that uses detailed nutritional, anthropometric, BIA, and sonographic data in combination in the same people. Since our study examined the relationship between AF and sarcopenia in a diverse spectrum of individuals, from apparently normal older adults to those with neurological disease, a broader generalization may be possible.

In conclusion, we found that muscle health in people with AF was less favorable than in those without AF, as indicated by clinical, ultrasound imaging, and bioimpedance parameters. Additional studies are now needed to examine this relationship in terms of risk management, pathophysiology, and possible treatment.

## Figures and Tables

**Figure:**
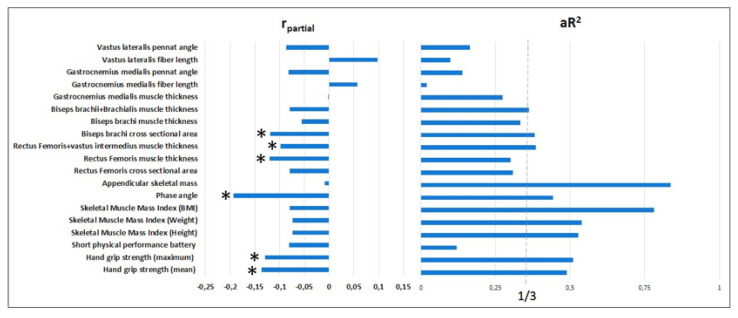
Partial correlation coefficients and determination coefficients. Footnote: Stars represent statistically significant values adjusted for age, female sex, height, and weight.

**Table 1:** Demographic characteristics and nutritional/sarcopenia indices in patients with and without AF.

	Atrial fibrillation (+)	Atrial fibrillation (−)	
N	72	538	**p**
**A. Demography/risk factors**			
Age	70.9 ± 10.5	66.2 ± 12.4	0.002
Female, %	49%	53%	0.481
Hypertension	82%	55%	<0.001
Diabetes mellitus	36%	27%	0.082
Dyslipidemia	31%	18%	0.010
Coronary artery disease	56%	23%	<0.001
Heart failure	25%	4%	<0.001
Rheumatic valve disease	1.4%	0.4%	0.241
Prosthetic heart valve disease	8.3%	1.6%	0.001
**B. Anthropometry**			
Height (cm)	161.3 ± 10	161 ± 10	0.813
Weight (kg)	77.9 ± 17	74.1 ± 14.8	0.051
BMI (kg/m^2^)	29.8 ± 5.5	28.8 ± 6.2	0.184
Waist circumference (cm)	100.5 ± 16.4	97.5 ± 13.7	0.104
Hip circumference (cm)	106 ± 11.8	103.8 ± 10.9	0.126
Thigh circumference right (cm)	53.2 ± 5.9	51.9 ± 5.6	0.205
Thigh circumference left (cm)	53.4 ± 5.9	51.9 ± 5.6	0.146
Calf circumference right (cm)	37.5 ± 3.8	36.4 ± 3.7	0.103
Calf circumference left (cm)	37.2 ± 3.7	36.4 ± 3.7	0.253
Calf average (cm)	37.4 ± 3.7	36.4 ± 3.7	0.163
Arm circumference right (cm)	31.7 ± 3.4	30.5 ± 3.7	0.079
Arm circumference left (cm)	31.8 ± 3.3	30.4 ± 4	0.050
**C. Performance**			
Short Physical Performance Battery	9.6 ± 2.9	9.9 ± 2.9	0.551
**D. Hand grip strength test**			
Hand grip strength average	19.9 ± 7.7	22.9 ± 8.6	0.163
Hand grip strength maximum	23.2 ± 8.3	26.2 ± 9.3	0.073
**E. Sarcopenia consequences**			
Morse Fall Scale	21.2 ± 17.3	17.9 ± 17.3	0.301
Fall Efficacy Scale	30.6 ± 33.4	20.1 ± 22.5	0.018
Barthel Index for ADL	95.9 ± 7.5	96.7 ± 8.9	0.656
Lawton and Brody Instrumental ADL	7 ± 1.9	7 ± 1.9	0.961
SarQol total score	64.7 ± 21.5	75.4 ± 18	0.002
Fatigue Severity Scale	4.6 ± 3	5.2 ± 11.5	0.752
Beck Depression Inventory	14.4 ± 12.4	10.4 ± 9.2	0.025
**F. Nutrition**			
MUST	0.5 ± 0.8	0.2 ± 0.6	0.053
FOIS	7 ± 0	7 ± 0.2	0.399
EAT-10	2.1 ± 4.6	1.5 ± 4.4	0.435

**Abbreviations:** ADL: Activities of Daily Living; EAT-10: Eating Assessment Tool; FOIS: Functional Oral Intake Scale; MUST: Malnutrition Universal Screening Tool; SarQol: Sarcopenia - Quality of Life

**Table 2:** Sarcopenia indices in patients with and without AF.

	Atrial fibrillation (+)	Atrial fibrillation (−)	P
n	72	538	
**A. Risk**			
SARC-F	2.5 ± 2.6	1.4 ± 1.9	0.003
**B. Ultrasonography**			
Biceps brachii cross-sectional area (cm^2^)	7.8 ± 2.8	8.8 ± 3.2	0.011
Arm anterior muscle (Biceps + Brachialis) thickness (mm)	21.8 ± 5.5	22.7 ± 5.4	0.179
Arm muscle thickness (mm)	22 ± 5.6	22.7 ± 5.3	0.318
Biceps brachii muscle thickness (mm)	16.4 ± 4	17.1 ± 4.4	0.246
Arm subcutaneous adipose tissue thickness (mm)	5.09 ± 2.64	4.36 ± 2.65	0.031
Rectus femoris thickness (mm)	9.7 ± 2.4	10.9 ± 2.9	0.002
Rectus femoris cross-sectional area (cm^2^)	4.7 ± 1.5	5.1 ± 1.6	0.054
Thigh Muscle Thickness (mm)	17.44±5.69	19.29±5.61	0.012
Thigh subcutaneous adipose tissue thickness (mm)	10.82 ± 4.96	10.34 ± 5.51	0.492
Vastus Lateralis muscle thickness (mm)	12.65 ± 2.7	13.11 ± 3.87	0.215
Vastus Lateralis Fascicle length (mm)	80.3 ± 19.8	74.1 ± 16.1	0.006
Vastus Lateralis pennation angle	9.6 ± 3.1	10.5 ± 3.1	0.030
Gastrocnemius medialis muscle thickness (mm)	15 ± 3.3	15.1 ± 3	0.703
Gastrocnemius medialis Fascicle length (mm)	39.2 ± 8	37.8 ± 8.2	0.180
Gastrocnemius medialis pennation angle	20.1 ± 5.2	21.9 ± 5.5	0.014
**C. Bioimpedance analysis**			
Skeletal Muscle Mass Index - SSMI(height)	10.7 ± 1.9	10.7 ± 1.7	0.733
Skeletal Muscle Mass Index - SMMI(weight)	36.1 ± 6.1	38.2 ± 6.1	0.009
Skeletal Muscle Mass Index - SMMI(BMI)	1 ± 0.2	1 ± 0.2	0.124
Appendicular skeletal mass - ASM	21.6 ± 4.9	20.8 ± 4.3	0.307
Phase Angle	4.6 ± 1	5.2 ± 1	<0.001
